# Ligaments: Their Nature and Morphology

**Published:** 1897-12

**Authors:** 


### Reviews of Books.

**Ligaments: their Nature and Morphology.** By J. Bland Sutton. Second Edition. Pp. 114. London: H. K. Lewis. 1897.

To the student who regards the word "ligament" with almost the same loathing as he does the word "bone" this book must be a revelation. Should he read this book—and we earnestly recommend him to do so—we feel confident that he will be induced to change his mind. He will no longer look upon a ligament as a band of fibrous tissue specially designed to trip him up in his examinations, but will be induced to ask himself—Has it not a nobler purpose? Instead of confining his attention to cramming up its position and its attachments, he will be compelled to think, and an enchanting vista will be opened up before him. He will unwittingly have been drawn into the meshes of that fascinating study, Comparative Morphology. The study of ligaments will be no longer a "bore," but a pleasure; and it is because this book may induce the student to think, that it meets with our heartiest approval. There are many fascinating chapters in the book; there is none more so than that concerning the history of the ligamentum teres of the hip-joint. Let the reader, after perusing this account, turn his attention to the many accounts given by various authors of the nature of this ligament, and contrast them; the conclusion is irresistible. We think he may with similar profit unravel the history of the superior gleno-humeral ligament.

These are but two examples, but they are enough to compel the reader to go farther, and when he has finished the book he will feel admiration for the talent and energy of the author who composed it.

